# Effects of resveratrol, granulocyte-macrophage colony-stimulating factor or dichloroacetic acid in the culture media on embryonic development and pregnancy rates in aged mice

**DOI:** 10.18632/aging.102768

**Published:** 2020-02-06

**Authors:** Jeong Yoon, Kyoung-Mi Juhn, Eun-Hye Jung, Hye-Jeong Park, San-Hyun Yoon, Yong Ko, Chang-Young Hur, Jin-Ho Lim

**Affiliations:** 1 Maria Research Center, Jungnang-gu, Seoul 02098, Korea; 2 Korea University, Seongbuk-gu, Seoul 02473, Korea; 3 Maria S Hospital, Jungnang-gu, Seoul 02098, Korea; 4 Maria Fertility Hospital, Dongdaemun-gu, Seoul 02586, Korea

**Keywords:** resveratrol, reproductive aging, pregnancy rate, mitochondria, ROS

## Abstract

The success rate of assisted reproductive technology is closely correlated with maternal age. Reproductive aging pathologies are frequently caused by impaired DNA repair, genomic instability, and mitochondrial dysfunction. Several reports have shown that resveratrol can prevent age-related diseases by improving mitochondrial function. Improved blastocyst development and mitochondrial output by dichloroacetic acid (DCA) supplementation were reported in aged mice. Granulocyte-macrophage colony-stimulating factor (GM-CSF) has significant effects on implantation rates in women with previous miscarriages. Therefore, this study was conducted to observe how those compounds influence the developmental and the reproductive potential of aged oocytes. BDF1 female mice at 58–62 weeks old were used for this study. MII oocytes were fertilized and cultured in MRC media supplemented with or without resveratrol (0.5 μM), GM-CSF (2 ng/ml) or DCA (1.0 mM). The addition of resveratrol, GM-CSF or DCA tended to increase blastocyst development and pregnancy rates. Supplementation with resveratrol significantly increased the pregnancy and implantation rates (*p* < 0.05). Moreover, resveratrol decreased reactive oxygen species production and increased mitochondrial membrane potential. These results suggest that the addition of resveratrol can increase pregnancy outcomes in women of advanced maternal age.

## INTRODUCTION

Due to recent lifestyle changes, many women are delaying childbearing and it is associated with an increased risk of infertility [1]. Previous studies have suggested that the reproductive capacity in women of advanced maternal age is significantly lower than that in women under 35 years of age [2–4]. The major factors in the reproductive capacity of older patients are the decrease in oocyte quality, low fertilization rate, poor embryonic development, low pregnancy rate, and increased rate of chromosomal aberrations. These can lead to increased aneuploidy, miscarriage, and birth defects [2, 5]. Reproductive aging pathologies are frequently associated with impaired DNA repair, metabolic disorders, genomic instability, telomeric shortening, apoptosis, and mitochondrial dysfunction [3, 6, 7]. However, various aspects of those pathologies require further studies because understanding the mechanisms of reproductive aging is essential to improving the pregnancy rates in advanced age women.

Resveratrol is a natural polyphenolic compound found in many plant species, such as grapes, peanuts, and berries [8, 9]. Resveratrol is known to possess biological activities, including antioxidant, anti-inflammatory, anti-carcinogenic, and telomerase-enhancing activities [8, 10]. In addition, several studies have reported that resveratrol can improve *in vitro* oocyte maturation (IVM) and embryonic developmental competence in various species [6, 11–13]. Furthermore, resveratrol can delay aging and prevent age-related diseases by improving mitochondrial function and decreasing reactive oxygen species (ROS) production [6, 8]. Liu et al. (2018) suggested that resveratrol can induce oocyte maturation and subsequent embryonic development in aged mice.

Granulocyte-macrophage colony-stimulating factor (GM-CSF) is a multifunctional cytokine expressed in the female genital tract during early pregnancy [14, 15]. The addition of GM-CSF to culture medium can promote preimplantation embryonic development and decrease apoptosis [14, 16]. GM-CSF supplementation can also improve pregnancy rates in patients with multiple unsuccessful *in vitro* fertilization (IVF) attempts and previous miscarriages [15, 17, 18]. Zhou et al. (2016) [19] reported that the addition of GM-CSF could decrease the occurrence of biochemical pregnancies in women aged over 35 years.

Dichloroacetic acid (DCA) is a stimulator of pyruvate dehydrogenase activity, thus accelerating the uptake of pyruvate into the tricarboxylic acid (TCA) cycle and increasing adenosine triphosphate (ATP) output [20, 21]. A previous study demonstrated that the addition of DCA to embryo culture media improved blastocyst development rates and mitochondrial output in embryos produced from aged mice [21].

With the recent trends of delaying marriage and childbirth, understanding the mechanisms occurring in oocytes and embryos in advanced maternal age is very important to improve pregnancy rates. However, only a limited number of studies have been conducted on pregnancy rates and embryonic development rates in older women. Therefore, the objective of this study was to determine the effects of resveratrol, GM-CSF or DCA in the culture media on embryonic development and pregnancy rates in aged mice.

## RESULTS

### Effect of different resveratrol concentrations on embryonic development

A dose-response trial was performed using young mice to determine the appropriate concentrations of resveratrol because different effective concentrations of resveratrol have been reported. Various resveratrol concentrations (0, 0.1, 0.5, 1.0, and 2.0 μM) were used to evaluate the effective concentrations for further experiments. The experiment was repeated four times in each group. There were no statistically significant differences in fertilization rates between the groups, although high concentrations of resveratrol tended to decrease fertilization rates (Table 1). The percentage of 4-cell stage embryos was not influenced by the resveratrol concentration. The blastocyst formation rate and hatching blastocyst rate on day 4 were higher in the group treated with 0.5 μM resveratrol than in the other groups. In addition, significant (*p* < 0.05) differences were observed between 0.5 and 2.0 μM (Table 1). Therefore, resveratrol was used at a concentration of 0.5 μM for all subsequent experiments.

**Table 1:** Effects of different concentrations of resveratrol on mouse embryonic development.

	**No. of oocytes**	**No. (%) of 2-cells**		**No. (%) of 4-cells <**		**No. (%) of embryos on day 4**							
						TBR^*^		**> Hatching**		**Expended**		**Degeneration**	
Control	121	115	(95.0)	115	(100.0)	107	(93.0^a^)	64	(55.7 ^ab^)	43	(37.4)	8	(7.0^a^)
0.1 μM	124	116	(93.5)	115	(99.1)	108	(93.1^a^)	74	(63.8 ^ab^)	34	(29.3)	8	(6.9^a^)
0.5 μM	127	121	(95.3)	121	(100.0)	115	(95.0^a^)	91	(75.2^a^)	24	(19.8)	6	(5.0^a^)
1.0 μM	120	108	(90.0)	104	(96.3)	93	(86.1^ab^)	54	(50.0 ^ab^)	39	(36.1)	15	(13.9^ab^)
2.0 μM	115	96	(83.5)	92	(95.8)	76	(79.2^b^)	36	(37.5^b^)	40	(41.7)	20	(20.8^b^)

*TBR - Total Blastocyst Rate

^a, b^ Different superscripts letters indicate significant differences within each column (*p* < 0.05)

### Effects of resveratrol, GM-CSF or DCA on fertilization and embryo development

MII oocytes from aged females were divided into four groups, fertilized and cultured in MRC media in the presence or absence of resveratrol (0.5 μM), GM-CSF (2 ng/ml) or DCA (1.0 mM). There were no statistically significant differences in fertilization rates between the groups assessed by successful completion of the first cleavage (control: 94.6%; resveratrol: 97.2%; GM-CSF: 96.2%; DCA: 97.4%) (Table 2). The addition of resveratrol, GM-CSF or DCA (92.4%, 92.1%, and 95.9%, respectively) tended to increase the total blastocyst formation rate at 96 hours of culture. However, only the DCA group had a significantly higher blastocyst formation rate than the control group (95.9% vs. 86.9%, *p* < 0.05) (Table 2).

**Table 2:** Effects of resveratrol, GM-CSF, or DCA on mouse embryonic development.

	**No. of oocytes**	**No. (%) of 2-cells**		**No. (%) of 4-cells <**		**No. (%) of embryos on day 4**							
						TBR^*^		**> Hatching**		**Expended**		**Degeneration**	
Control	186	176	(94.6)	172	(97.7)	153	(86.9^a^)	132	(75.0)	21	(11.9)	23	(13.1^a^)
Resveratrol	177	172	(97.2)	171	(99.4)	159	(92.4^ab^)	140	(81.4)	19	(11.0)	13	(7.6^ab^)
GM-CSF	158	152	(96.2)	151	(99.3)	140	(92.1^ab^)	123	(80.9)	17	(11.2)	12	(7.9^ab^)
DCA	152	148	(97.4)	148	(100.0)	142	(95.9^b^)	116	(78.4)	26	(17.6)	6	(4.1^b^)

*TBR - Total Blastocyst Rate

^a, b^ Different superscripts letters indicate significant differences within each column (*p* < 0.05)

### Effects of resveratrol, GM-CSF or DCA on pregnancy and implantation rates

To determine whether resveratrol, GM-CSF or DCA could improve the pregnancy and implantation rates, differently treated blastocysts were transferred into the uterus of pseudo-pregnant female mice. After blastocyst transfer to recipient females, pregnancy and implantation rates in the GM-CSF (56.3% and 26.1%) and DCA (60.7% and 22.1%) groups were increased compared to those of the control group (48.4% and 19.0%), although the differences were not statistically significant (Table 3). In contrast, supplementation with resveratrol significantly increased the pregnancy rate compared to the control group (*p* < 0.05). After 246 blastocysts of the resveratrol group were transferred to 29 pseudo-pregnant recipients, 22 pregnancies were achieved (22/29, 75.9%) (Table 3). Furthermore, the implantation rate assessed by the number of live pups per transferred embryo was significantly higher in the resveratrol group (87/246, 35.4%) than in the control group (51/268, 19.0%) (*p* < 0.05). After birth, all live pups were morphologically normal. These results indicate that supplementation of the medium with resveratrol effectively enhanced the post-implantation development of embryos in aged mice.

**Table 3:** Live pups produced following the transfer of blastocysts derived from each group.

	**No. of recipients**	**No. of pregnant recipients**	**Pregnancy rates**	**No. of embryos transferred**	**No. of live pups**	**Implantation rates**
Control	31	15	48.4^a^	268	51	19.0^a^
Resveratrol	29	22	75.9^b^	246	87	35.4^b^
GM-CSF	32	18	56.3^ab^	276	72	26.1^ab^
DCA	28	17	60.7^ab^	240	53	22.1^a^

^a, b^ Different superscripts letters indicate significant differences within each column (*p* < 0.05)

### Effect of resveratrol supplementation on intracellular ROS levels in embryos from aged mice

**Figure 1:**
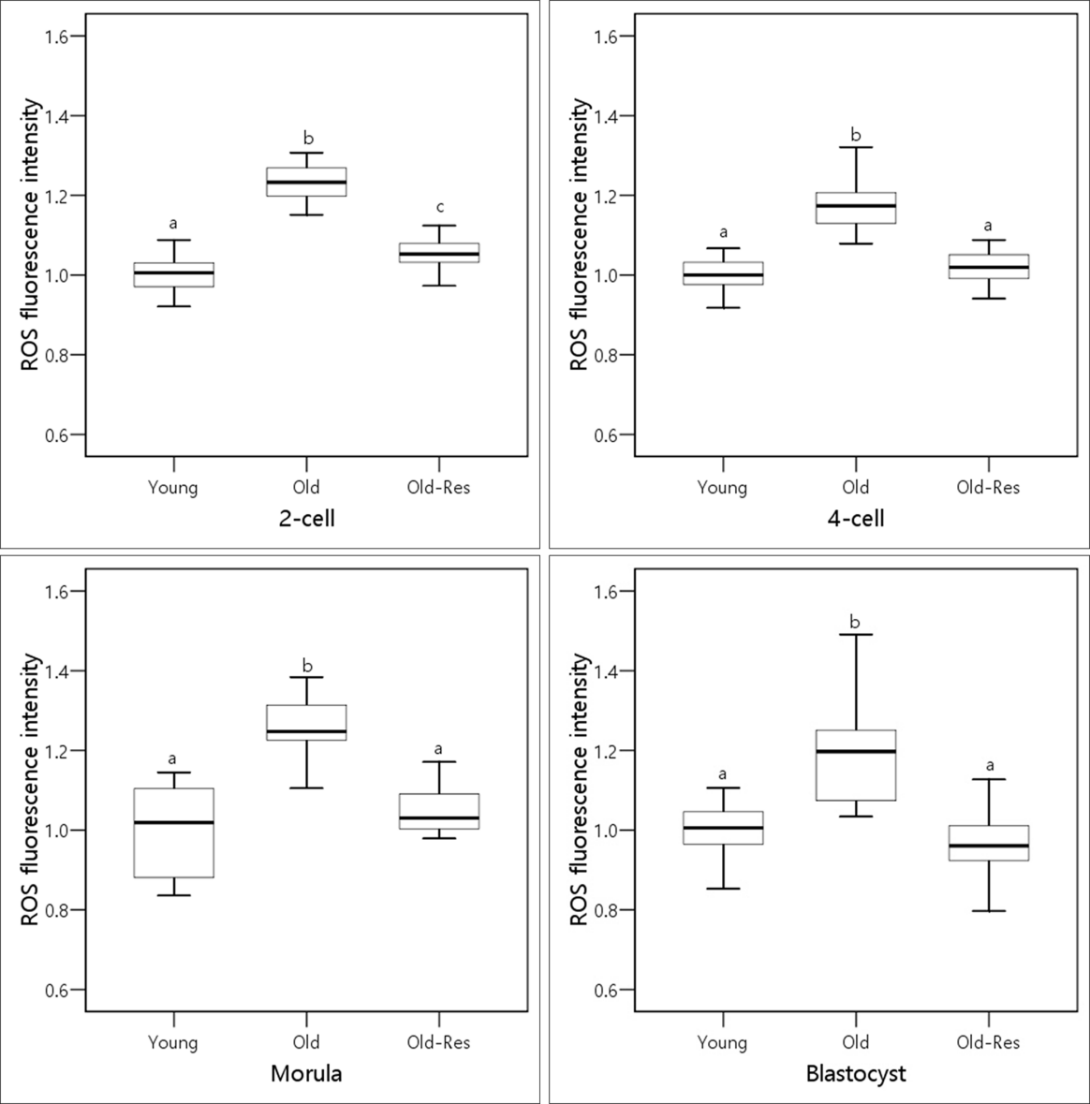
**Boxplots representing the effect of resveratrol supplementation on ROS levels.** ^a,b,c^Different superscripts indicate significant differences within a stage (*p <* 0.01).

To determine whether resveratrol might protect against oxidative stress, embryos from each group were stained with dichlorodihydrofluorescein diacetate (DCFH-DA) to quantify the intracellular ROS levels (Figure 1). The mean fluorescence intensity of ROS in the embryos of the old group was about 1.2-fold higher (*p* < 0.01) than that of the young group. Resveratrol treatment significantly (*p* < 0.01) decreased the intracellular ROS levels throughout the pre-implantation embryonic stages in aged mice. These results suggest that resveratrol could decrease ROS levels induced by aging.

### Effect of resveratrol supplementation on MMPs of embryos from aged mice

**Figure 2:**
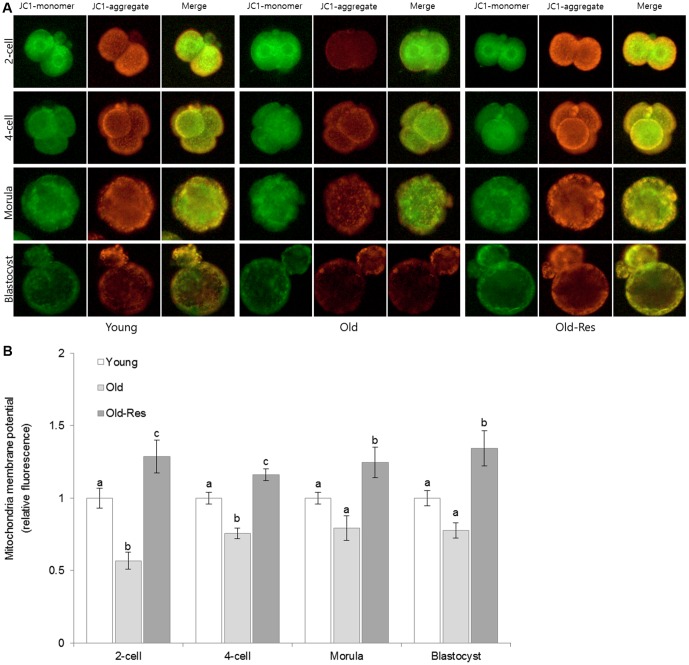
**Effect of resveratrol supplementation on mouse embryonic mitochondrial membrane potential.** (**A**) Representative pictures stained with JC-1. Green and red fluorescence indicate JC-1 monomers and JC-1 aggregates, respectively. (**B**) The ratio of red:green JC-1 fluorescence in embryos. The data are presented as the mean ± SEM from three independent experiments. ^a,b,c^ Different superscripts indicate significant differences within a stage (*p <* 0.05).

To further elucidate the positive effects of resveratrol on pregnancy and implantation rates, the mitochondrial membrane potentials (MMPs) were examined (Figure 2). The average MMP in the young group was higher than that in the old group, with statistically significant differences at the 2-cell and 4-cell stages between the two groups (*p* < 0.05). Resveratrol supplementation increased the MMP levels throughout the pre-implantation embryo stages in aged mice. These increases were statistically significant in both young and old groups (p < 0.05).

## DISCUSSION

The success rate of assisted reproductive technology is correlated with maternal age, which affects pregnancy and miscarriage rates [7, 22]. Studies have reported that reproductive capacity and oocytes were affected by old age, resulting in poor quality oocytes and mitochondrial dysfunction [2, 6, 23].

Oocyte aging is divided into two physiological processes, postovulatory aging and maternal aging [22]. Oocyte aging associated with maternal aging is a major problem in most clinical laboratories. Therefore, it is important to establish a protocol to improve both the developmental rate of oocytes and the pregnancy rate in response to oocyte aging due to maternal aging in *in vitro* fertilization programs. Since oocytes at ovum pick-up are already in the MII stage, studies on mitosis rather than meiosis may be more effective in improving the pregnancy rate in older patients. Mitochondrial dysfunction and ROS production during mitosis in aged embryos were reported to influence the developmental competence of pre-implantation embryos [22]. Therefore, this study was conducted to observe how selected compounds influenced the developmental and reproductive potentials of aged oocytes.

In contrast to GM-CSF or DCA, reports of the effective resveratrol concentrations for embryonic development have varied depending on the study group, animal species, and culture medium used [10, 12, 13, 24]. Therefore, a dose-response trial was conducted to determine the appropriate concentration of resveratrol. High concentration (2.0 μM) significantly reduced the total blastocyst formation rate compared to the control or low concentrations (Table 1). This was consistent with previous studies indicating that high concentrations of resveratrol might have negative effects on embryonic development [25, 26]. However, since different results have been reported, differences in the fertilization method, the culture medium used, and the mice species might be associated with such discrepancies [10].

In older organisms, increased ROS levels, decreased mitochondrial functions, and/or increased miscarriage rates are frequently observed. Thus, three biomolecules (resveratrol, GM-CSF, and DCA) selected based on previous reports were tested for their effects on embryonic development and pregnancy rates in aged mice (Tables 2, 3). Trials with human embryos have shown that the addition of GM-CSF to culture media positively influenced the blastocyst development rate and implantation competence [15, 16]. Particularly, it has been shown that GM-CSF supplementation can improve pregnancy rates in patients with multiple unsuccessful IVF attempts and previous miscarriages [15, 17, 18]. In contrast, GM-CSF did not show any significant influence on embryonic developmental competence and pregnancy rates in this study, consistent with previous mice studies [27, 28]. This conflict might be explained by the studies of Elaimi et al. [28], who reported high proportions of chromosomal abnormalities in mouse blastocysts cultured in GM-CSF-containing media. This result suggests a reason why GM-CSF had no significant impact in our study. Different mechanisms between patients with previous miscarriages and older patients with high miscarriage rates might also affect the results. In addition, GM-CSF may not have a positive effect to decrease the abortion rate caused by aneuploidy in older women. This is, in part, consistent with the results reported by Zhou et al. [19]. They suggested that low concentrations of GM-CSF supplementation (0.2 ng/ml) in the culture medium had no beneficial effect on embryo quality, pregnancy rate, implantation rate, and the take-home baby rate of women aged over 35. To clarify the controversial results of the effects of GM-CSF on the embryonic developmental competence and pregnancy potential of older mice and humans, comparative studies of the GM-CSF mechanism of action are in progress.

McPherson et al. [21] reported that DCA supplementation stimulated mitochondrial activity, which in turn, increased the blastocyst development rates, fetal development, and MMP in mice aged 26–28 weeks. They concluded that stimulation of the mitochondria by DCA could improve embryos from aged mothers. Similarly, the present study found that the addition of DCA to the culture media significantly increased blastocyst formation compared to the control. However, DCA did not significantly affect the implantation rate (Table 3). These results are inconsistent with the above study, which reported that the fetal development of the DCA group (46.3%) was significantly higher than that of the control group (35.2%). These differences may be attributed to differences in species or the age of the mice, fertilization method, or culture medium used. Thus, further experimentation is necessary to determine the exact effect of DCA on aged mice.

In contrast to the effects of GM-CSF and DCA, resveratrol treatment significantly increased the pregnancy and implantation rates (Table 3), although it did not affect the fertilization and blastocyst development rates (Table 2). Several reports have suggested that resveratrol can delay aging and prevent aging-related diseases by improving mitochondrial function and decreasing ROS [6, 8]. Therefore, to examine the mechanism behind these results, the effect of resveratrol on ROS expression and mitochondrial function in aged mice embryo was investigated**.**

ROS are normally generated through oxidative phosphorylation in the mitochondria [29, 30]. However, high levels of ROS can damage many cellular biomolecules, such as carbohydrates, lipids, proteins, and DNA, which is thought to be involved in aging-related pathologies [22]. Bentov et al. [23] and Silva et al. [29] reported that mitochondrial DNA mutations and mitochondrial dysfunction increased with maternal aging and that ROS levels in oocytes from older women were associated with declines in oocyte and embryo quality. Resveratrol has beneficial effects on embryonic development by decreasing ROS levels [25, 31–35]. Therefore, the present study investigated whether resveratrol could increase the pregnancy and implantation rates in aged mice by reducing ROS levels (Figure 1). During pre-implantation development, ROS levels in the embryos of old mice were about 1.2-fold higher than those of young mice. This indicates that elevated ROS levels in embryos from old mice might contribute to the low pregnancy rates. The addition of resveratrol significantly reduced ROS levels in the embryos of older mice to levels similar to those of young mice. This result is, in part, consistent with the results of Liang et al. [11]. They reported that the addition of resveratrol increased the resistance of post-ovulatory aging and decreased ROS levels. Abe et al. [25] also suggested that blastocysts developed after resveratrol treatment showed low ROS levels. These results imply the positive effects of resveratrol on embryonic development and pregnancies in aged mice.

Mitochondria are the major source of energy in all eukaryotic cells. It has been suggested that mitochondrial dysfunction results in chromosomal anomalies during mitosis, influencing the developmental competence of pre-implantation embryos [22, 29, 30]. Mitochondrial functions depend on the maintenance of membrane potential [2]. Thus, the effect of resveratrol on mitochondrial functions was evaluated by measuring MMP levels. The MMPs in young mice embryos were higher than those in old mice embryos (Figure 2). Resveratrol supplementation significantly increased MMPs throughout stages of pre-implantation embryos in old mice, compared to those in the embryos of young mice. These results suggest that the increased pregnancy and implantation rates after resveratrol supplementation were, in part, due to higher MMP.

Several studies have reported a correlation between increased MMPs in early-stage embryos (1~2 cell) and improved embryonic development [21, 36]. Particularly, Silva et al. [29] demonstrated that supplementation with antioxidants could increase membrane potentials in 2-cell embryos of aged mice and that this increase was correlated with embryonic development. In the present study, MMP increased by resveratrol treatment was 2.3-fold higher in the 2-cell stage than in any other stage (4-cell: 1.5-fold, morula: 1.6-fold, blastocyst: 1.7-fold, compared to old mice embryos). These results indicate that the large increase in MMP during the early embryonic stage might have affected the developmental competence of embryos and the pregnancy rate in aged mice.

In addition to the ROS levels and MMP, histone deacetylation can also explain how resveratrol upregulates pregnancy rates. Resveratrol is known to be a SIRT1 activator, which is involved in the aging process through histone deacetylation [35, 37]. Reports showing undetectable SIRT1 protein in old oocytes and increased maternal age-related aneuploidy by decreased histone deacetylation suggest that advanced maternal age is related to SIRT1 expression and histone deacetylation [4, 38, 39]. They also suggest the possibility that resveratrol may improve the embryonic development and pregnancy rates of aged mice by increasing SIRT1 and stabilizing histone deacetylation. This was supported by the results of Liu et al. demonstrating that resveratrol supplementation in IVM medium increased SIRT1 mRNA expression in oocytes from aged mice [8]. To investigate other mechanisms of action of resveratrol, experiments regarding SIRT1 expression and histone deacetylation are in progress.

In conclusion, the current study was the first to investigate the effects of physiological regulators (GM-CSF, DCA, and resveratrol) on the developmental competence and pregnancy potential of aged mice embryos. Among the three substances, resveratrol significantly improved pregnancy and implantation rates. These results are thought to be due to the biological activities of resveratrol as an anti-oxidant and/or a mitochondrial nutrient. The results from more extensive studies of the physiological and molecular mechanisms of resveratrol may increase the successful pregnancy rates in older patients.

## MATERIALS AND METHODS

### Ethics approval

All animal procedures were performed in compliance with the NIH Guide for the Care and Use of Laboratory Animals. Animal studies were approved by the Animal Research Ethics Committee at the Maria Research Center (Permit number: 2018-002).

### Experimental design

Experiment 1: The effects of resveratrol, GM-CSF or DCA on the development of embryos in aged mice were examined.

Experiment 2: The effects of resveratrol supplementation in *in vitro* culture on the ROS levels and MMP of embryos from aged mice were examined.

### Oocyte collection

B6D2 F1 (C57BL/6×DBA) female mice at 58–62 weeks of age were used for this study. As a control, 6-8-week-old female mice were used for the young group and the dose-response experiments. The old group of mice received 10 IU of gonadotropin while the young group of mice received 5 IU. Each female mouse was given an intraperitoneal injection of pregnant mare serum gonadotropin (PMSG; Folligon, Intervet, UK) followed by an injection of human chorionic gonadotropin (hCG; Chorulon, Intervet, UK) 48 h later. The mice were euthanized by cervical dislocation and the metaphase II (MII) oocytes were collected 14 h post-hCG injection in pre-warmed MRC#D01 medium (Maria Medical Foundation, South Korea) as described previously [40].

### *In vitro* fertilization and culture

*In vitro* fertilization was conducted in MRC#D01 medium using sperm collected from the cauda epididymis of B6D2 F1 males in MRC#D01 medium. Five hours after the insemination, zygotes were cultured sequentially in cleavage and blastocyst medium (MRC#D13 and 46; Maria Medical Foundation, South Korea) for 96 h as described previously [40]. In both phases, the embryos were cultured in groups of 10 embryos per 50 μl drop of culture media under mineral oil. The oocytes from aged females were divided into four groups, fertilized and cultured in MRC media in the presence or absence of resveratrol (0.5 μM; Sigma-Aldrich, USA), GM-CSF (2 ng/ml; Invitrogen, USA), or DCA (1.0 mM; Sigma-Aldrich). The concentration of resveratrol used in this experiment was determined by a dose-response trial. Concentrations of 2 ng/ml for GM-CSF and 1.0 mM for DCA were chosen based on previous reports [15, 21].

### Embryo transfer

Embryo transfer was carried out as described previously [41]. Briefly, CD-1 females were allowed to mate with vasectomized males to induce pseudo-pregnancy. Successful mating was confirmed by a copulation plug check the following morning. The pseudo-pregnant mice at 2.5 days post-coitum (dpc) were randomly assigned to four groups. Prior to embryo transfer (ET), the recipients were anesthetized by placing in a chamber filled with 2–3% isoflurane for 2–3 min (Hana Pharm, South Korea) in oxygen. ET was carried out using a non-surgical embryo transfer device (NSET) (ParaTechs Co., USA) according to the manufacturers’ instructions. The recipients were allowed to deliver at term to evaluate the pregnancy and implantation rates. The pregnancy rate was calculated as the number of pregnant mice as a proportion of the total number of transferred mice. The implantation rate was calculated as the number of live pups as a proportion of the total number of embryos transferred.

### Detection of intracellular ROS levels

To examine the difference in ROS levels between the young and old mice embryos, young mice embryos were used as positive controls. ROS levels within the embryos were measured using a DCFH-DA (Thermo Fisher Scientific, Inc., USA). The embryos were incubated in 10 μM DCFH-DA at 37 °C for 30 min and then washed three times in phosphate-buffered saline (PBS). The embryos were mounted onto glass slides and observed under a fluorescence microscope (Nikon, Japan). The fluorescence intensities in each embryo were quantified using Image J.

### Measurement of MMP

MMP was measured using a 5,5’,6,6’-tetrachloro-1,1’,3,3’-tetraethylbenzimidazolylcarbocyanine iodide (JC-1) fluorophore (Thermo Fisher Scientific, Inc., USA). The embryos were cultured with 2 μM JC-1 staining solution at 37°C for 30 min and then washed three times with PBS. For the control samples, the embryos were incubated with 10 μM carbonyl cyanide 3-chlorophenylhydrazone (CCCP) at 37 °C for 20 min and then stained with JC-1. The fluorescence intensity was detected at different developmental stages (2-cell, 4-cell, morula, and blastocyst) by using a fluorescence microscope (Nikon, Japan). Upon labeling with JC-1, the embryos with polarized mitochondria emitted a red fluorescence signal (J-aggregate), whereas the embryos with depolarized mitochondria emitted a green fluorescence signal (J-monomer). The ratio of J-aggregates to J-monomers was then calculated.

### Statistical analyses

All experiments were repeated at least three times. Comparisons of data among the groups were performed with one-way analysis of variance (ANOVA) followed by Duncan post-hoc examinations. The pregnancy rates were compared using Chi-squared tests. The data were analyzed using the SPSS Statistical Package version 17 (SPSS Inc., Chicago, IL, USA). A *p*-value of less than 0.05 was considered to indicate statistical significance.
